# Editorial: Epidemiology, evidence-based care, and outcomes in spinal cord injury

**DOI:** 10.3389/fneur.2024.1383757

**Published:** 2024-03-04

**Authors:** Nader Fallah, Vanessa K. Noonan, Lisa N. Sharwood

**Affiliations:** ^1^Praxis Spinal Cord Institute, Vancouver, BC, Canada; ^2^Department of Medicine, University of British Columbia, Vancouver, BC, Canada; ^3^University of New South Wales, Sydney, NSW, Australia; ^4^University of Technology, Ultimo, NSW, Australia

**Keywords:** epidemiology, outcomes, translation, trauma, motor neuron disease of disease, spinal cord injury

This edition of Frontiers in Neurology is dedicated to sharing progress in the epidemiology, evidence-based practice, and outcomes of spinal cord injury (SCI). The collection is divided into two parts: the first part describes the epidemiology of SCI, and the second part explores evidence-based approaches to care and patient outcomes. The increasing use of artificial intelligence and analytical methods such as machine learning, is also highlighted.

Spinal cord injury is a life altering condition that has a profound effect on an individual's motor, sensory and autonomic functions which impacts their ability to participate in society and can decrease their quality of life. With an aging population, there is a need to understand the epidemiology and economic implications as we plan for the future. Spinal cord injury interventions must be evaluated for effectiveness – not just economically, but to measure their impact on outcomes that are important to individuals living with SCI (such as neurology, function, mortality, and quality of life). Effective interventions and targeted implementation are required to ensure that individuals and the healthcare system benefit.

## Epidemiology of SCI

The first part of this Focus Issue includes six articles that describe the epidemiology of SCI in both pediatric and adult populations. There is also a article on the burden of Motor Neuron Disease.

In the first article by Thorogood et al. the incidence and prevalence of traumatic SCI were assessed for the Canadian population using ICD-10 codes. The reported incidence in 2019 was 1,199 cases (32/million), and the prevalence was 30,239. The study introduced a standardized method for calculating the incidence and prevalence of traumatic SCI in Canada through national-level health administrative data. Despite acknowledging the conservative nature of the estimates due to data limitations, the study represents a substantial Canadian sample over a 15-year period, providing insights into national trends.

Hu et al. undertook a systematic review and meta-analysis of epidemiology of traumatic SCI (from 1978 to 2022), including a total of 59 reports from 23 provinces in China. The random pooled incidence of traumatic SCI in China was reported as 65.15 per million population with a range of 6.7 to 569.7 per million population in their meta-analysis.

The co-occurrence of traumatic brain injury (TBI) and SCI, often termed “dual diagnosis,” presents clinical and rehabilitation complexities. Gober et al. conducted a study with the aim of assessing the point prevalence of comorbid TBI among children hospitalized with SCI between 2016 and 2018 from U.S. hospitals participating in the Kids' Inpatient Database. Their findings revealed that 38.8% of children admitted with SCI also had a comorbid TBI. The study concluded that comorbid TBI is prevalent among United States children experiencing SCI, emphasizing the need for further research to better understand the impact of dual diagnosis on mortality, quality of life, and functional outcomes.

Jiang et al. conducted a study on the epidemiological characteristics of traumatic SCI in China, providing insights into the incidence, prevalence, and external causes. They determined that the point prevalence of traumatic SCI, standardized to the China census population of 2010, was 569.7 per 1,000,000 in the general population, 753.6 per 1,000,000 among men, and 387.7 per 1,000,000 among women. Additionally, the reported annual incidence of traumatic SCI was 49.8 per 1,000,000 in the overall population, 63.2 per 1,000,000 among men, and 36.9 per 1,000,000 among women. The study estimated a total of 759,302 prevalent cases of traumatic SCI and identified 66,374 new traumatic SCI cases annually in China for 2010.

Very little is known about the epidemiology of pediatric SCI. The study published by Crispo et al. is therefore an important contribution to fill this knowledge gap, reporting the annual rate of pediatric emergency department visits for traumatic SCI using the Healthcare Cost and Utilization Project -Nationwide Emergency Department Sample. The study found that the annual emergency visit rate has remained stable between 2016 and 2020, with ~2,200 new all-cause pediatric emergency department visits with a diagnosis of traumatic SCI annually and that cervical injuries were most prevalent. Their findings also suggested that the proportion of sports-related traumatic SCI emergency department visits increased recently.

Park et al. assessed the global burden of MND from 1990 to 2019 as part of the Global Burden of Disease, Injuries, and Risk Factor study. They included various MNDs, including amyotrophic lateral sclerosis, progressive muscular atrophy, primary lateral sclerosis, pseudobulbar palsy, spinal muscular atrophy, and hereditary spastic paraplegia. The estimates indicated ~63,700 incident cases annually and 268,673 prevalent cases of MND worldwide. In 2019, MND resulted in 39,081 deaths globally. The age-standardized rates for MND incidence, prevalence, and mortality in 2019 were calculated at 0.79 per 100,000 people, 3.37 per 100,000 people, and 0.48 per 100,000 people, respectively.

## Evidence-based care and outcomes following SCI

The second section of this Focus Issue includes articles on evidence-based care and outcomes following SCI. It has been reported that it takes an average of 17 years for research evidence to be translated into practice, which highlights the importance of timely knowledge translation (1, 2).

Previously, several articles have been published (3–5) to develop a clinical algorithm for use in the acute care setting to predict the probability that an individual will be able to walk at 1-year post injury. Hakimjavadi et al. described their efforts in launching a website (https://www.ambulation.ca/) to make an ambulation tool accessible to the public, and actively monitor end-user feedback to enhance its usability for the future. This tool serves as a valuable step in bridging the gap between knowledge and impact for clinicians, persons living with SCI and families and can serve as a model for other clinical algorithms.

The design and analysis of clinical trial data is challenging due to the heterogeneity of the injury (6). A solution to address this challenge is to identify similar subgroups based on patient demographics and baseline injury characteristics. Basiratzadeh et al. applied machine learning methodology to establish a more homogeneous group, illustrating how these patient subgroups could effectively discern differences in outcomes.

Furthermore, SCI studies often have small sample sizes due to the low incidence, resulting in under-powered results that can lead to inconclusive findings. This challenge can be addressed by optimizing the study design. Fallah, Noonan, Waheed et al. highlight the importance of stratifying or using an appropriate control group to obtain accurate conclusions about a treatment efficacy (in randomized controlled trial or observational studies), particularly when dealing with small sample sizes. This study demonstrates the importance of recording the baseline neurological examination date and time and ensuring the control and intervention groups are well-matched.

The Standing and Walking Assessment Tool (SWAT) serves as a standardized objective staging tool used in Canada to assess lower limb function in individuals with traumatic SCI. The use of SWAT was investigated in individuals with non-traumatic SCI or disease by Alavinia et al.. Specifically, the research aimed to evaluate the convergent validity of SWAT for inpatients with non-traumatic SCI. The study concluded that SWAT demonstrates sufficient evidence for convergent validity and responsiveness in persons with non-traumatic SCI or disease, making it a valuable tool for describing standing and walking recovery.

Physical activity among individuals with SCI is often decreased following injury. Olsen et al. assessed the intervention ProACTIVE SCI, evaluating its reach, effectiveness, adoption, implementation, and maintenance. This intervention led by physiotherapists and SCI peer coaches during the rehabilitation-to-community transition, successfully reached the majority of patients and has the potential to increase physical activity following SCI.

As the population ages, multi-morbidity is becoming a growing health concern, and individuals with SCI often have pre-existing comorbidities prior to their injury. The health conditions (comorbidities and secondary complications following SCI) can lead to increased healthcare utilization and diminished health outcomes. Fallah, Hong et al. utilized network models, a form of machine learning, on the Canadian SCI Community Survey dataset (7). They adapted the original 30 item Multi-morbidity Index (MMI) and created a concise version of the index, called the MMI-25. Their results demonstrated that multi-morbidity in persons with SCI is associated with higher healthcare utilization, as well as lower levels of physical and mental health and quality of life.

The global prevalence of people with disabilities is estimated to surpass 1 billion (2017), with over half residing in low- and middle-income nations (8). Cui et al. conducted a review encompassing the epidemiological features of stroke and SCI. They described the therapeutic outcomes and recent advancements in the utilization of both conventional and innovative orthotic devices for both stroke and SCI.

Finally, Fallah, Noonan, Thorogood et al. explored the association between body mass index (BMI) measured after acute traumatic SCI and the impact on mortality. Their study yielded two noteworthy findings. First, a higher BMI was identified as a mild protective factor linked to lower mortality in individuals with SCI, aligning with a modest “obesity paradox” that has been reported in health conditions such as stroke (9). Conversely, being underweight emerged as a significant risk factor for death during acute care and up to 7 years post-SCI. Second, the study did not use the World Health Organization criteria designed for able-bodied individuals, since it was found to have limitations for persons with SCI. The researchers employed a data-driven approach to define BMI ranges associated with distinct mortality risks following SCI. Future work will include understanding the underlying mechanisms and validating these results in other studies.

## Author contributions

NF: Writing—original draft, Writing—review & editing. VN: Writing—original draft, Writing—review & editing. LS: Writing—review & editing.
